# Expiratory Flow – Vital Capacity: Airway – Lung Dysanapsis in 7 Year Olds Born Very Preterm?

**DOI:** 10.3389/fphys.2018.00650

**Published:** 2018-05-29

**Authors:** Iulia Ioan, Aurore Gemble, Isabelle Hamon, Cyril Schweitzer, Stéphanie Metche, Claude Bonabel, Phi L. Nguyen-Thi, Jean-Michel Hascoet, Silvia Demoulin-Alexikova, François Marchal

**Affiliations:** ^1^Department of Pediatric Respiratory Function Testing, Children’s Hospital, Vandoeuvre-lès-Nancy, France; ^2^EA 3450 DevAH-Department of Physiology, Faculty of Medicine, University of Lorraine, Nancy, France; ^3^Department of Pediatrics, Children’s Hospital, Vandoeuvre-lès-Nancy, France; ^4^Department of Neonatal Medicine, Regional Maternity Hospital, Nancy, France; ^5^Department of Epidemiology, Faculty of Medicine, University of Lorraine, Nancy, France

**Keywords:** premature birth, lung function, school age, airway-lung size, forced expiration

## Abstract

An index normalizing airway dimension for lung size derived from spirometry was found inversely correlated to lung size in school children born very preterm, indicating larger alveolar volumes draining into comparatively smaller airways. In contrast in children born full term the index was independent of lung size.

## Introduction

Follow up studies of children born very preterm (VP) are pointing at compromised lung function outcome (Korhonen et al., 2004; Fawke et al., 2010) extending further to respiratory sequelae at adult age (Vrijlandt et al., 2006; Narang et al., 2008; Wong et al., 2008; Aukland et al., 2009; Doyle et al., 2017). In addition to chronic lung disease (CLD) in the neonatal period/early infancy (Aukland et al., 2009; Brostrom et al., 2010; Hacking et al., 2013; Vom Hove et al., 2014; Fortuna et al., 2016) low birth weight and/or low gestational age have been reported to be associated with poor lung function in childhood (Vrijlandt et al., 2006; Fawke et al., 2010; Lum et al., 2011; Cazzato et al., 2013; Hacking et al., 2013; Fortuna et al., 2016; Simpson et al., 2017). Postnatal alveolarization has been described non-invasively in healthy subjects throughout childhood and adolescence (Narayanan et al., 2012) and has also been identified around puberty as a catch up phenomenon in those born VP (Narayanan et al., 2013). Therefore, conducting airways may adjust to alveoli during development differently in school children born VP and those born full term (FT).

During a forced expiration, the dynamic airway compression limits flow as a function of the resistance of that part of the airway tree upstream to the equal pressure point. The maximum expiratory flow at 50% of the forced vital capacity (MEF50) for instance, may be thus taken as an index of airway dimension, while the forced vital capacity (FVC) is proportional to lung size. Therefore the congruence between lung size and airway dimension may be studied during that particular situation where intrathoracic airways are dynamically compressed. Mead proposed the MEF50/FVC ratio as an index of airway size relative to lung size, and a simple diagram to functionally assess the dependence of the former to FVC (Mead, 1980). School age is an interesting period to study lung function outcome in children born prematurely because reliable spirometry may readily be obtained in most instances, the lung still undergoes important processes of development and, hopefully in the future, appropriate therapeutic interventions at this age may become efficient in minimizing later respiratory sequelae.

The aim of the study was to describe the relationship between MEF50/FVC and FVC in a cohort of school children born VP followed up at 7 years (Hamon et al., 2013) with reference to age matched healthy controls born FT. It was thought that studying in a narrow age interval prior to puberty would provide a sharp window for more precise functional assessment of airway dimension relative to lung size. The straight forward assumption was that – should airway size be proportional to lung size – the ratio MEF50/FVC would show little systematic trend when plotted against FVC. The null hypothesis was that – in a tight age interval – both groups would show airway dimensions proportional to lung size. Because the main interest was – as much as possible – focused on the effect of being born VP, the population was selected to include only patients that did not present with immediate acute severe respiratory distress at birth.

## Materials and Methods

### Subjects

A prospective follow up study of management of early, severe hypoxemic respiratory failure in VP included a reference group with mild or no neonatal respiratory distress (Hamon et al., 2005). This group underwent lung function response to exercise at 7 years (Hamon et al., 2013) and the corresponding baseline data serve as a basis for the current analysis. Durations of oxygen and mechanical ventilatory support, administration of surfactant and incidence of CLD (Allen et al., 2003) had been documented from neonatal records. Age-matched healthy children born at term were recruited from local primary schools during the same period (Marchal et al., 2008). The medical record included items relative to atopy, asthma and maternal smoking status. All children were free of respiratory symptoms at time of the testing. They underwent standardized clinical examination and pulmonary function testing. Both patient and control group criteria included a medical history negative for atopy and asthma. Written informed consents were obtained from the children and their parents at the time of lung function testing.

The study was approved by the Ethics Committee (Comité de Protection des Personnes de Lorraine) and registered with the ClinicalTrials.gov registry (no. NCT00390065).

### Measurements

Spirometry was measured according to current standards (Miller et al., 2005; Beydon et al., 2007). FVC and FEV1 were expressed as *Z*-scores using recently published algorithms (Quanjer et al., 2012). MEF50 was extracted from the data base to compute the MEF50/FVC ratio. The study group also underwent plethysmographic measurements of static lung volume as part of their routine assessment, and the residual volume to total lung capacity ratio taken as an index of lung distension, when larger than the upper limit of predicted 95% confidence interval (Stocks and Quanjer, 1995).

### Statistical Analysis

The SAS 9.4 Package was used. Continuous variables were expressed as mean and standard deviation. Group comparisons were performed using *t*-test, analyses of variance or chi-squared test as needed. The relationship of the type:

$$MEF50/FVC=a*{FVC}^{b}$$

was tested by linear regression of the corresponding log transforms and the significance assessed by Pearson’s correlation coefficient. The effect of independent variables on MEF50/FVC was assessed using multiple factorial analysis. A *p*-value less than 0.05 was retained as statistically significant.

## Results

The neonatal characteristics, demographics at school age and lung function data of the 46 VP and 27 FT are presented in **Table 1**. Age and height were similar between groups but maternal smoking during pregnancy was more frequent in patients (*p* = 0.02). FEV1 – *Z*-score but not FVC – *Z*-score was significantly lower in preterm (respectively, *p* = 0.022 and *p* = 0.37, **Table 1**) and the trend for a difference in FEV1/FVC was not significant (*p* = 0.08, **Table 1**).

**Table 1:** Characteristics of the children.

	Control	Preterm	*p*
*n* (M/F)	27 (9/18)	46 (19/27)	
Age (year)	7.0 ± 0.5	7.2 ± 0.4	0.14
Height (cm)	121.8 ± 5.9	119.8 ± 5.7	0.17
Weight (kg)	24.3 ± 3.5	22.5 ± 3.3	0.026
GA (week)	39.7 ± 1.2	28.9 ± 1.8	0.000
BW (kg)	3.26 ± 0.42	1.19 ± 0.34	0.000
Surfactant (n)		28	
MV (day)		28 ± 29	
O_2_ (day)		42 ± 73	
CLD (n)		7	
Pregnancy smoking	3/25	11/27	0.02
Current smoking	5/27	18/45	0.06
FVC-Z	0.43 ± 0.79	0.2 ± 1.19	0.37
FEV1-Z	0.84 ± 0.95	0.27 ± 1.05	0.022
MMEF-Z	0.16 ± 0.93	-0.6 ± 1.1	0.004
FEV1/FVC	0.94 ± 0.04	0.91 ± 0.08	0.08
RV/TLC	-	0.23 ± 0.09*	
MEF50/FVC	1.35 ± 0.83	1.16 ± 0.40	0.018


*GA, gestational age; BW, birth weight; MV, mechanical ventilation; CLD, chronic lung disease; FVC, forced vital capacity; FEV1, forced expiratory volume in 1s; MMEF, maximum mid-expiratory flow; RV, residual volume; TLC, total lung capacity; MEF50, maximum expiratory flow at 50% FVC. ^∗^*n* = 43.*

**FIGURE 1:**
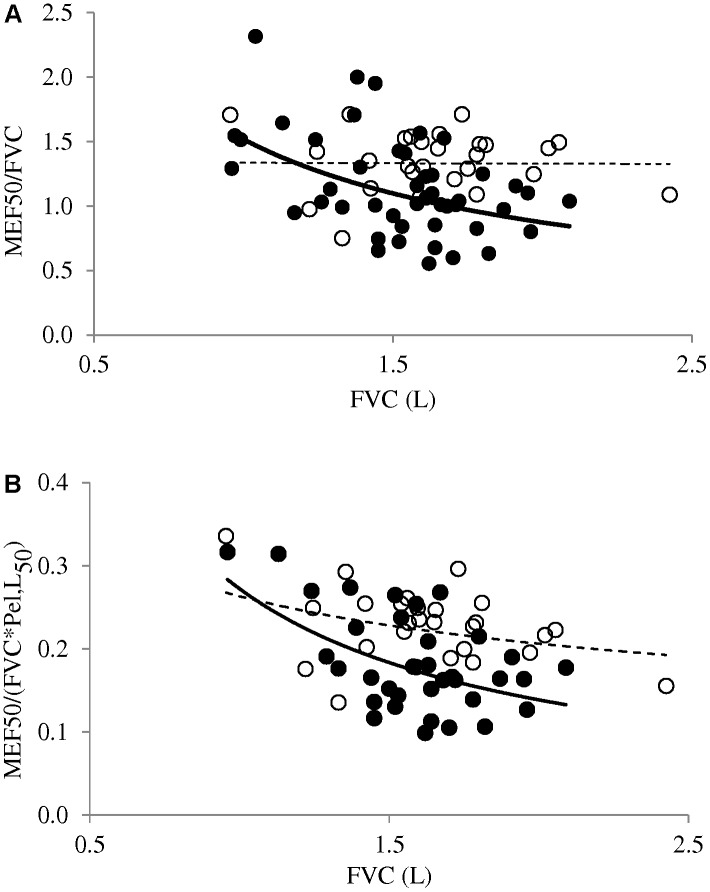
**(A)** The MEF50 to FVC ratio is independent of FVC in children born at term (open symbols, *p* > 0.1), but is negatively correlated to FVC in those born extremely preterm (closed symbols, *p* < 0.005). **(B)** MEF50/FVC divided by the estimated elastic recoil pressure at corresponding volume (Pel,L_50_) shows relationships to FVC similar to **(A)**, with similar differences between controls and patients (only non-distended children are taken into account, see section “Discussion”).

The MEF50/FVC ratio was lower in VP than FT (*p* = 0.018), with no difference between girls and boys (*p* = 0.34) and no dependence on either pregnancy- or on current maternal smoking (*p* > 0.1). MEF50/FVC was related neither to surfactant administration at birth (*p* = 0.53), nor to occurrence of CLD (*p* = 0.9). When scattering the MEF50/FVC vs. FVC data points, a striking difference was observed between VP and FT. FT’s displayed the expected independence of MEF50/FVC on FVC, that is, the MEF50 increased in proportion with FVC, with the ratio being constant throughout the FVC interval studied, and no significant correlation was observed (*p* > 0.1, **Figure 1A**). In contrast, VP’s showed a significant correlation between MEF50/FVC and FVC (*p* < 0.005, **Figure 1A**) and the negative slope indicated that larger FVC’s were associated with comparatively smaller airways. Plethysmographic measurements were obtained in 43 VP (**Table 1**) and the RV/TLC ratio larger than 0.33 suggested lung distension in 8 according to current standards (Stocks and Quanjer, 1995).

## Discussion

This study indicates constant MEF50/FVC in a range of FVC in healthy children aged 6–8 years, while the ratio is correlated to FVC in children born VP. There is thus a strong suggestion that adjustment of airway dimension to lung size is different in children born VP and those born FT. At 7 years, the latter show airway size adjusted to lung size, while in the former, the negative slope is in keeping with larger alveolar volumes draining into comparatively smaller airways. The lung function picture is consistent with alveolarization pursuing on top of a damaged conducting airway tree. The observation is in keeping with alveolarization pursuing well beyond the neonatal period reported in the experimental animal (Kovar et al., 2002) as well as in healthy teenagers (Narayanan et al., 2012) and fits the recent report in prematurely born children at adolescence (Narayanan et al., 2013).

The precise cause for the current observation may be questioned: is it immature lung development or a consequence of the initial respiratory condition and ensuing complications? The very immature lung usually goes through a variety of insults resulting from acute neonatal respiratory distress, breathing high oxygen concentration, pulmonary infection, and barotrauma with prolonged mechanical ventilation. Those factors known to be implicated in the development of CLD are thus frequently associated with evidence of airway obstruction on spirometry at school age (Korhonen et al., 2004). Not unexpectedly, children with CLD frequently show lower lung function at school age, not only with reference to healthy controls born full term, but also with their peers without CLD (Fawke et al., 2010). To minimize as much as possible this contribution in our analysis, only those patients not presenting with severe, acute respiratory distress at birth were included (Hamon et al., 2005). Surfactant administration in this context was not found to impact on the MEF50/FVC. While a few children eventually developed CLD, the condition was not found to be a significant determinant to the MEF50/FVC at school age. Therefore prematurity *per se* – a major determinant to lung function outcome in childhood (Chan et al., 1989) – most likely accounted for the difference between FT and VP described in **Figure 1**. The condition of prematurity of course includes the deleterious effect of early exposing the immature airways to room air, and prenatal factors such as exposure to tobacco smoke (Stocks and Dezateux, 2003) that was found more frequent in our study group but not directly associated with MEF50/FVC.

For a more precise analysis of airway – alveolar adjustment in conditions of flow limitation, it was suggested to account for the elastic lung recoil (Pel,L) (Mead, 1980), which opposes – in a volume-dependent manner – the dynamic airway compression and is therefore another determinant for maximum expiratory flow (Pride et al., 1967). The dysanaptic ratio divides MEF50/FVC by the Pel,L at 50% of the FVC [MEF50/(FVC^∗^Pel,L_50_)] – which has dimensions of specific conductance upstream to the choke point (Mead, 1980). Therefore our interpretation of MEF50/FVC as an index of correspondence between lung size and airways dimensions depends on Pel,L_50_ comparability between VP and FT. Lung distension has been reported at school age in some children born prematurely (Simpson et al., 2017) and was suggested here in eight children from the VP group where RV/TLC was larger than the upper limit of the 95% confidence interval (Stocks and Quanjer, 1995). A decreased Pel,L_50_ in those subjects could result, as shown from prior measurements in mild emphysematous adult lungs (Duke et al., 2018). A proper estimation of Pel,L would have required invasive procedures, not appropriate in this context. Instead, the height – based prediction equation of Pel,L_50_ described in healthy children (Zapletal et al., 1976) was used to derive a dysanaptic ratio in our control subjects and in those patients where no overt increased RV/TLC was identified. It was thus found that neither the negative relationship observed in VP, nor the lack of significant slope in FT described in **Figure 1A** would be altered when MEF50/FVC was changed for the dysanaptic ratio (**Figure 1B**). It is also worth mentioning that, in the original study of Mead, the inter-subject variability was found to be decreased when MEF50/(FVC^∗^Pel,L_50_) was substituted to MEF50/FVC, but the finding of a negative slope vs. FVC was unaltered in that adult population aged 23–48 years (Mead, 1980).

The pattern described in **Figure 1** implies that a significant positive regression exists between maximal expiratory flow and FVC in healthy children, but not in children born EP. In fact, a significant power relationship has previously been shown between maximal mid expiratory flow and FVC in healthy children in cross sectional studies at different ages (Merkus et al., 1993), as well as with longitudinal measurements during growth (Martin et al., 1988). Such positive relationship could also be extracted from those prediction equations against height, presented by Wang et al. (1993) for healthy children aged 7 or 8 years. The same positive relationship could be extracted from our control group, in contrast with children born VP who did not show such significant regression (not shown for clarity).

We believe the current information adds to the description of lung function characteristics of school children born VP. We speculate that longitudinal data on MEF50/FVC and dysanaptic ratio may prove to be important indicators to the long term outcome of airway – lung size matching, as suggested by a recent investigation of young adults born VP (Duke et al., 2018). In line with previous suggestions (Silverman and Kuehni, 2007; Stern et al., 2007; Narang, 2010; Stocks et al., 2013) the data also raise the possibility that prematurity in general could account for some unexpected spirometry findings at adult age. For instance, the report of asymptomatic adult subjects presenting with reduced FEV1/FVC but normal FEV1 (Barisione et al., 2009) questions the possibility of dysanaptic lung growth, possibly in relation to a (forgotten) history of premature birth. Finally the study suggests that maximal expiratory flows derived from the late part of the flow volume loop – that have not proven useful so far in the routine pediatric lung function testing – may eventually show some role in the pathophysiological assessment of airway-lung development.

## Author Contributions

II, IH, CB, CS, J-MH, SD-A, and FM have prepared the project of this study. II, IH, CB, CS, SD-A, and FM managed preparatory phase of the study. IH, J-MH, CS, CB, SD-A, and FM performed participant recruitment. II, CS, CB, and FM performed lung function tests. AG, II, IH, CB, CS, J-MH, SD-A, PN-T, and FM performed data collection and statistics. II, AG, SM, CS, J-MH, SD-A, and FM have prepared the draft of manuscript. II, AG, SM, PN-T, J-MH, CS, SD-A, and FM completed the work and revised the final manuscript.

## Conflict of Interest Statement

The authors declare that the research was conducted in the absence of any commercial or financial relationships that could be construed as a potential conflict of interest.
